# Temporal and cultural limits of privacy in smartphone app usage

**DOI:** 10.1038/s41598-021-82294-1

**Published:** 2021-02-16

**Authors:** Vedran Sekara, Laura Alessandretti, Enys Mones, Håkan Jonsson

**Affiliations:** 1 Sony Mobile Communications, 22188 Lund, Sweden; 2 Department of Computer Science, IT University of Copenhagen, Copenhagen S, 2300 Denmark; 3 Department of Applied Mathematics and Computer Science, Technical University of Denmark, 2800 Kongens Lyngby, Denmark; 4 Copenhagen Center for Social Data Science, University of Copenhagen, Copenhagen K, 1353 Denmark; 5 Faculty of Engineering (LTH), University of Lund, 22100 Lund, Sweden

**Keywords:** Information theory and computation, Applied mathematics

## Abstract

Large-scale collection of human behavioural data by companies raises serious privacy concerns. We show that behaviour captured in the form of application usage data collected from smartphones is highly unique even in large datasets encompassing millions of individuals. This makes behaviour-based re-identification of users across datasets possible. We study 12 months of data from 3.5 million people from 33 countries and show that although four apps are enough to uniquely re-identify 91.2% of individuals using a simple strategy based on public information, there are considerable seasonal and cultural variations in re-identification rates. We find that people have more unique app-fingerprints during summer months making it easier to re-identify them. Further, we find significant variations in uniqueness across countries, and reveal that American users are the easiest to re-identify, while Finns have the least unique app-fingerprints. We show that differences across countries can largely be explained by two characteristics of the country specific app-ecosystems: the popularity distribution and the size of app-fingerprints. Our work highlights problems with current policies intended to protect user privacy and emphasizes that policies cannot directly be ported between countries. We anticipate this will nuance the discussion around re-identifiability in digital datasets and improve digital privacy.

## Introduction

Tracking behaviour is a fundamental part of the big-data economy, allowing companies and organizations to segment, profile and understand their users in increasingly greater detail^1^. Modeling context and interests of users has proven to have various commercial advantages: products can be designed to better fit customer needs; content can be adapted; and advertising can be made more relevant^2–5^. Efficient user modeling requires the collection of large-scale datasets of human behaviour, which has led to a growing proportion of human activities to be recorded and stored^6^. Today, most of our interactions with computers are stored in a database, whether it is an e-mail, phone call, credit-card transaction, Facebook like, or online search, and the rate of information growth is expected to accelerate even further in the future^7^. These rich digital traces can be compiled into detailed representations of human behaviour and can revolutionize how we organize our societies, fight diseases, and perform research; however, they also raise serious privacy concerns^8–13^.

The sensing capabilities of modern day smartphones, together with our seemingly symbiotic relationship to them, render mobile devices good tools for tracking and studying human behaviour^14,15^. Mobile phones are ubiquitous and have permeated nearly every human society. In 2018 there were 107 mobile-cellular subscriptions per 100 inhabitants^16^, and globally smartphones account for 60% of all mobile phones^17^. Smartphones have transformed the way people access the internet; today the majority of traffic to web pages stems from mobile devices rather than from desktop computers^18^, making advertisers target mobile phones to a higher degree. With the standard methods based on cookies for identifying customers not being used in smartphones, combined with the rising usage of ad-blockers among users^19^, companies, advertisers, and so-called *data brokers* are now using smartphone apps to identify and track individuals. Identifiers such as the Android Advertisement ID are one example of IDs embedded in app, but they do not allow data brokers to track users across multiple applications or devices, and they can even be reset by users. App usage behaviour, however, cannot be cleared, and it is hard (and in many cases not feasible) to be changed or manipulated by users.

This creates an economic incentive for global population tracking of app usage. In fact, many free apps are developed for the sole purpose of data collection^20^, and numerous apps have been shown to collect information about installed (or used) apps which is sent to third-party data brokers^21^. Although app usage information might seem innocent it is highly unique and can be used to predict sensitive individual characteristics such as age, ethnicity, income, religion or relationship status^22,23^. A recent report, released by the U.S. Federal Trade Commission, describes how data broker companies obtain vast amounts of personal data, which they further enrich with additional online and offline sources, and re-sell these improved datasets to the highest bidder, typically without the explicit consent or knowledge of users^24^. An example of this is TalkingData, China’s largest third-party mobile data platform, which collects and sells app usage data of more than 750 million smartphone users^25^.

According to U.S. privacy laws, data is considered anonymous if it does not contain personally identifiable information (PII) such as name, home address, email address, phone number, social security number, or any other obvious identifier. As a result, it is legal for companies to share and sell anonymized versions of a dataset. Similarly, to comply with EU’s General Data Protection Regulation (GDPR) it is common practice for companies to encrypt the user identifier and throw away the key, while keeping the actual user data. However, as studies have shown, the mere absence of PII in a dataset does not necessarily guarantee anonymity due to the fact that it is relatively easy to compromise the privacy of individuals by using outside information^8,11,26^.

Human behaviour, although imbued with routines, is inherently diverse. For instance, previous work (on datasets of limited size—approximately 50.000 users and without accounting for geographic spread) has shown that 99.4% of smartphone users have unique app usage patterns and established the viability of using apps as markers of human identity, similar in application to fingerprints in forensic science^27^.

Our study looks into uniqueness on a much larger scale—3.5 million individuals—and focuses on the smartphone apps used by these individuals. First, we use our dataset to validate the findings of Achara et al.^27^ demonstrating that even in a large-scale dataset it is relatively easy to uniquely identify individuals from only a handful of their apps. Next, we study the role played by seasonal and cultural differences in determining uniqueness, thus bringing more nuance to the discussion around re-identification. Our results show that human behaviour is easier to re-identify during certain periods of the year, and that country-specific factors influence the re-identification rate of individuals. Depending on the attack model, we identify that one of two quantities, namely the parameter characterizing the distribution of app-popularity within a given country and the median size of individuals’ app-fingerprints, to a large extent explains differences in re-identification rates across countries.

## Results

### Global uniqueness of human behaviour

**Figure 1:**
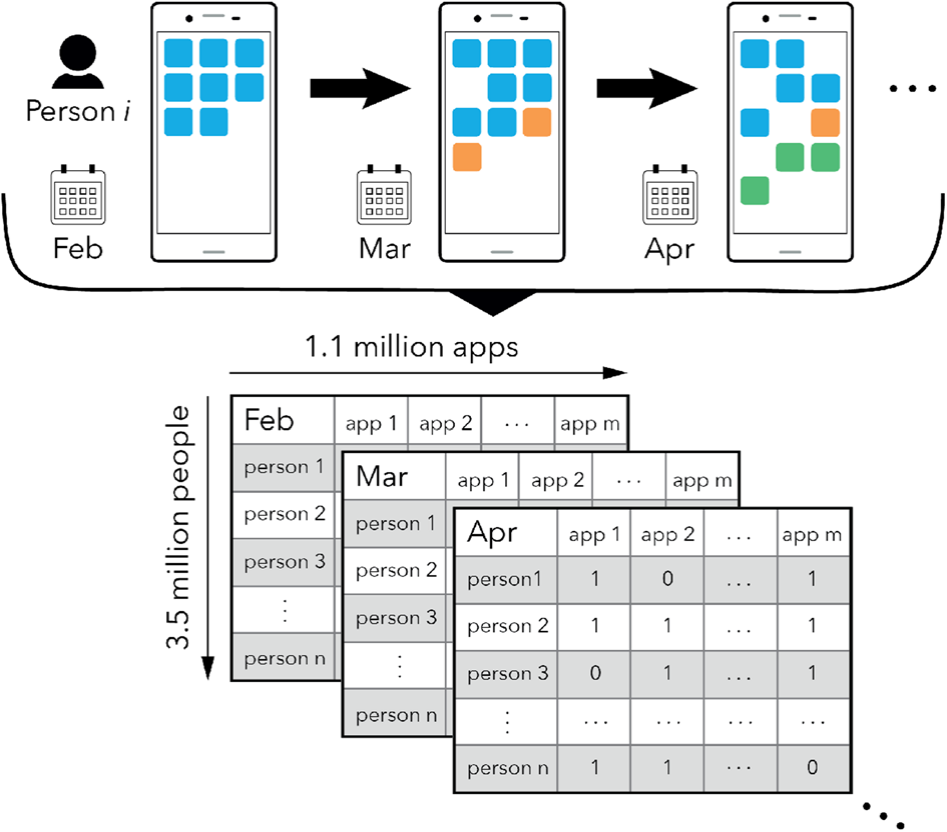
Smartphone app usage patterns change over time. This study is based on smartphone app-fingerprints of 3,556,083 individuals from 33 countries. For each month between February 2016 and January 2017, we retrieve the list of apps a person has used during the period. On average a person uses $n_{\text {month}} = 23$ unique apps per month, however, individuals continuously revise which apps they use, and use $n_{\text {year}} = 76$ apps over the course of a year. App-fingerprints are represented as a sparse *user* $\times$ *app* $\times$ *month* tensor, where 1 indicates a person has used an app during a specific month, 0 otherwise. To look at longer time-windows, we aggregate entries according to a maximum value heuristic and retain entries if they are greater than zero.

We evaluate the likelihood of identifying individuals within smartphone app usage data using a dataset that spans 12 months (1 Feb 2016 to 31 Jan 2017) and encompassing 3.5 million people from 33 countries using in total 1.1 million unique apps (approximately 40% of all apps on Google Play^28^). We have chosen to disregard phone vendor specific apps, such as alarm clock apps, built-in phone dialer apps, and only focus on apps that are downloadable from Google Play. From this we form app *fingerprints* for each person, i.e. a binary vector containing information about the apps a person has used for every month. We only consider apps actually used by a user and disregard apps that were installed but never used. Figure 1 illustrates the typical patterns of app usage, with individuals continuously changing their app-fingerprint over the course of a year by trying out new apps and ceasing to use others. As such, app-fingerprints slowly drift over time, with the average rate of change being roughly constant between consecutive months (Fig. S1). Although app-fingerprints drift, the number of apps people use on their smartphones is constant over time suggesting that humans have a limited capacity for interacting, navigating, and managing the plethora of services and social networks offered by smartphones (Fig. S2). This limiting effect has been observed in app usage^29^ as well as other aspects of life such as interactions among people^30^ and geo-spatial exploration^31^.

To estimate the re-identifiability of individuals, we focus on two attack schemes: (1) the *random attack scheme* and (2) the *popularity attack scheme*. These attack schemes relate to different threat models, where an attacker may try to associate two digital identities by comparing a dataset with outside information. Under the (1) random attack scheme, the assumption is that an attacker has information about *n* apps selected at random from the pool of an individual’s mobile phone apps. This is not a realistic scenario, since users typically share more information, but it provides a useful baseline. Under the (2) popularity attack scheme, an attacker would know the *n* least popular app used by a person. Although this is an equally unrealistic scenario, the analysis provides an upper bound to the estimation of uniqueness and highlights the need of better privacy guards against collection of app-usage data.

The risk of re-identifying individuals is estimated by means of unicity^11,12,27^, which is a standard method for quantifying how many points of outside information, on average, are needed to re-identify an individual (see Methods for details). Here, re-identification corresponds to a successful assignment of an app-fingerprint to a single unique individual in our dataset. This does not entail that we can directly get the *real* identity of a person, such as name, address, e-mail, social security number, etc. But this becomes possible if the information is cross-referenced with other data sources, which there unfortunately have been countless examples of^8,32–35^. Given an individual’s app-fingerprint, unicity quantifies the number of apps needed to uniquely re-identify that person; the fewer apps we need the more unique a person is and vice versa. Given a dataset of app-fingerprints and apps *i*, *j* and *k*, a person *u* is uniquely identifiable if they, and only they, in the dataset have used apps *i*, *j* and *k*, i.e. matching the fingerprint of user *u*. In our dataset we evaluate uniqueness as the percentage of individuals we can re-identify using *n* number of apps.

**Figure 2:**
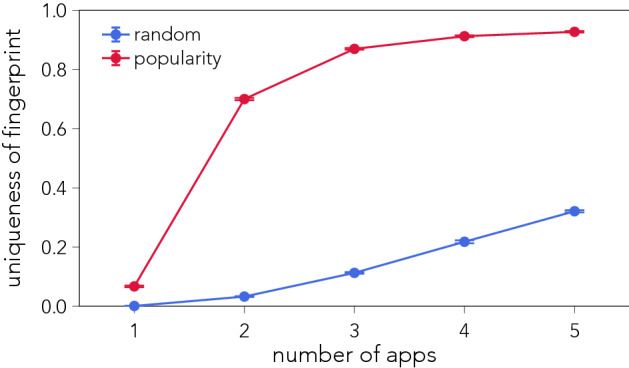
Uniqueness of smartphone app-fingerprints given *n* number of apps. Selecting apps at random is not an efficient way of identifying individuals and achieves a modest re-identification rate of $21.8\%$ when using 4 apps (blue line). Sampling apps according to their inverse popularity, in terms of users, yields significantly higher rates of re-identifications, $91.2\%$ when using 4 apps (red line). Error bars denote one standard deviation. App-fingerprints are constructed from the full 12 months of data, and $99.7\%$ of individuals within the dataset have a unique fingerprint.

Figure 2 shows the efficiency of the random attack scheme, demonstrating that 21.8% of individuals can be re-identified from 4 apps. A surprisingly high number given that we only use binary features (that is, has the user used the app or not) and not information regarding *when* an app was used or for *how long*—features which would only make fingerprints more unique. The popularity attack scheme is more effective at re-identifying individuals. Using just 2 apps the popularity approach greatly outperforms the random strategy, and using 4 apps, we are able to re-identify 91.2% of individuals. (This result confirms and is comparable to earlier findings which were derived from a much smaller and non-representative sample of Android users^27^.) The popularity scheme is more effective because it exploits the fact that app popularity follows a heavy-tailed distribution (see Fig. S3), where a few apps are used by millions of individuals, while an overwhelming majority of apps only have a couple of users. As such, the popularity scheme is more likely to pick apps which are highly unique (used by one or very few individuals). Information about app popularity is easily attainable, it can directly be estimated from the data, be scraped from *Google Play*, or even be purchased from data vendors.

### Temporal variability of anonymity

**Figure 3:**
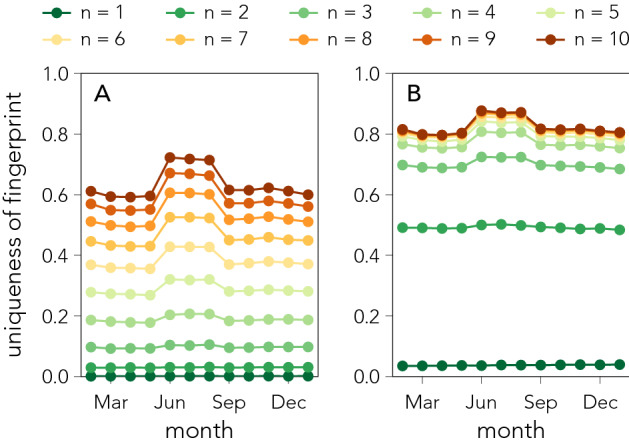
Temporal variations of uniqueness over 12 months. The fraction of individuals which we can re-identify by using *n* apps (1–10) changes from month to month, revealing that uniqueness has a temporal component, and that people are more unique during certain months. This is independent of whether apps are selected according to the random attack scheme (**A**) or the popularity scheme (**B**). Compared to Fig. 2, the fraction of re-identified individuals per month is lower because we have segmented behaviour into monthly fingerprints as compared to constructing fingerprints from 12 months of data. Each data point is rescaled proportional to the number of apps in each month so that uniqueness values are comparable even with a changing size of app set (see Fig. S5).

Human lives, routines, and behaviours evolve over time^31,36,37^, therefore individual app-fingerprints might become harder (or easier) to identify. To quantify the temporal variability of uniqueness, we construct monthly fingerprints for all individuals and evaluate anonymity on a monthly basis using the unicity framework. Figure 3 shows the fraction of individuals that are re-identifiable per month, and reveals an increased fraction of re-identifications for June, July, and August. The increase in uniqueness is independent of whether apps are sampled at random (Fig. 3A) or by popularity (Fig. 3B), and is statistically significant (see Fig. S6). For the random attack scheme people’s app-fingerprints are respectively $14.8\%$ and 18.4% more unique (4.1 and 11.2 percent point higher) when using 5 and 10 apps. In terms of the number of individuals this means approximately 145,000 ($n=5$) and 400,000 ($n=10$) people in our dataset adopt a behavior which makes them identifiable during summer. For the popularity strategy, we observe re-identification rates to be $8.8\%$ and $10\%$ higher (5.4 and 6.4 percent point) when using 5 and 10 apps, corresponding to 190,000 and 230,000 additional individuals being re-identified.

The increase in identifiability stems from a combination of related behavioural changes (Fig. S4). Apps related to categories such as “*travel*”, “*weather*”, “*sports*”, and “*health and fitness*” gain popularity during the summer months (June, July, August), related to people traveling and downloading apps that help them navigate new cities, use fitness apps to get into better shape, and start using apps that enable them to follow global sports events such as the 2016 UEFA European Championship. Simultaneously, usage of apps related to categories such as “*education*” and “*business*” becomes less popular. This suggests an interplay between people’s physical behaviour and their app-fingerprint, indicating that when people change their routines by traveling and exploring new places, they also change their app usage. This change in phone behaviour makes app-fingerprints more unique, and leads to higher re-identification rates.

### Cultural aspects of privacy

A natural question is whether individuals are more or less identifiable across different cultural contexts. Since our dataset includes information about individuals’ country of residency, we investigate the matter by computing the fraction of unique individuals within each of the countries in our database. To exclude effects due to country size we consider samples of equal size for each country (see Methods).

**Figure 4:**
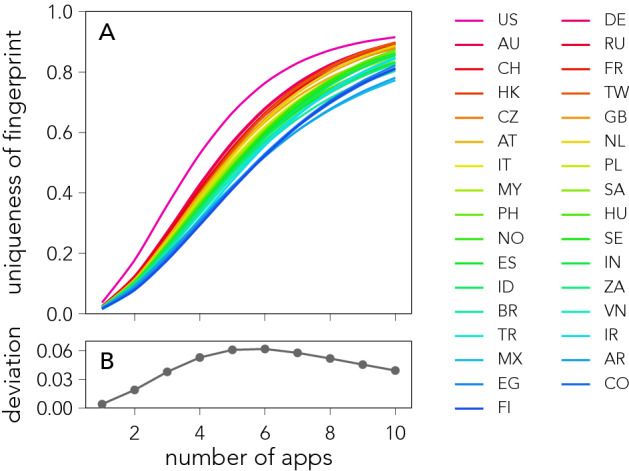
Identifying fingerprints across cultural contexts. (**A**) The average fraction of unique individuals within samples of 20,000 individuals from the same country, as a function of the number of apps *n* included in the fingerprint. Results are averaged across 240 samples, 20 for each of the months considered. Each line correspond to a different country. Countries in the legend are sorted based on the identification rate obtained for $n=5$, from highest to lowest. Re-identification rates are higher than in Fig. 2 as fingerprints are segmented into countries. (**B**) The standard deviation $\sigma$ for the number of unique individuals across countries, as a function of the number of apps included in the fingerprint.

Figure 4 shows that the country of residence has a large effect on the re-identification rate, even for the random attack scheme. Considering $n = 5$ apps, the average re-identification rate varies drastically between countries, from $(41.2 \pm 0.6) \%$ for Finland to $(66.5 \pm 0.5) \%$ for the United States (see Fig. 4A). Country differences are stable with respect to both time (see Fig. S9) and sample size (see Fig. S10), and the difference in uniqueness between any two countries is statistically significant for over $90\%$ of country pairs (see Fig. S11). Differences are also robust when selecting apps using the popularity scheme (see Fig. S12). However, the standard deviation ($\sigma$) between re-identification rates across different countries is not constant, it peaks for $n=6$ (see Fig. 4B), implying that differences between countries are more pronounced in an intermediate regime where the size of the app fingerprint is large enough but not as large as to include the majority of people’s apps (see Fig. S2)—a regime in which the unique number of individuals saturates.

The origin of the observed differences in re-identification rates between countries can result from a combination of factors including societal characteristics, adoption of internet and communication technologies, socio-economic conditions, etc. Among these factors, an important role could be played by the characteristics of the app-ecosystem. For instance, the probability *P* that an app is installed by a fraction of users *p* is well described by a power-law $P(p) \sim p^{- \beta }$ in all the countries under study (see De Nadai et al. for details^29^). The exponent $\beta$, however, varies greatly across countries, ranging from $\beta = 1.74 \pm 0.02$ in Finland to $\beta = 1.84 \pm 0.01$ in the United States (Fig. S13). (The exponents are relatively stable over time (see Fig. S14).) A large value of $\beta$ results in a skewed distribution, implying that app popularity is unevenly distributed, with a small fraction of apps being used by an overwhelming majority of individuals.

We test the extent to which the characteristics of the app-ecosystem explain re-identifaction rates using a linear regression model of country unicity as a function of three variables endogenous to the app-ecosystem of each country (number of apps, the median number of apps per person, and the slope of the app popularity distribution) and two control variables (internet adoption rates, and the Gini index of the wealth distribution). To explore monotonic relationships that are not necessarily linear we perform a rank transformation of all variables, such that rank 1 is the highest possible value of each variable^38^.

**Figure 5:**
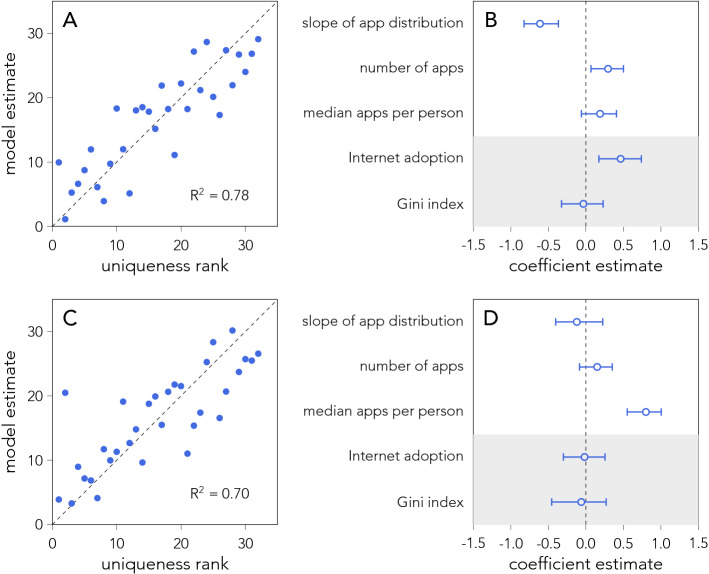
Estimating country uniqueness as function of multiple variables. (**A**) Model predictions using a linear model with rank transformed variables. Uniqueness values are estimated by randomly sampling 5 apps from individuals’ app-fingerprints. (**B**) Coefficient estimates of the model, excluding intercept, including 95% confidence intervals estimated from 10.000 bootstrap samples. The grey shaded area indicates control variables. (**C**) Model predictions for the attack scheme using $n=5$ apps. (**D**) Coefficient estimates for the attack scheme model with 95% confidence intervals.

Figure 5A shows the quality of the model fit, comparing country values of uniqueness (estimated using random sampling of $n=5$ apps, see Fig. S18 for different values of *n*) to model predictions (see SI section 3.1 for details). Remarkably, the model explains country uniqueness well ($R^2=0.78$). Figure 5B shows the coefficient estimates with 95% confidence intervals (see Methods). The most informative variable is the exponent of the app popularity distribution ($\beta$ in $P(p) \sim p^{- \beta }$), which is negatively associated with uniqueness (see also Fig. S19). Countries with more skewed app popularity distributions have higher uniqueness values (easier to identify people). As the app distribution is heavy-tailed, the more skewed the distribution becomes the more apps will exist in the long tail. As such, because we sample apps at random we are more likely to encounter an unpopular app. Further, the number of apps present in the ecosystems also has a small positive effect on the uniqueness, the more apps there are, the easier it is to randomly pick an app which makes a person unique. Interestingly, one of the control variables, internet adoption, is positively associated with uniqueness. (Similarly, GDP is correlated with uniqueness ($r = 0.64$, $p < 10^{-4}$), but is not included in the model due to high collinearity with internet adoption, see SI Section 3.1 for details.)

Similar to Fig. 5A, we have built a model to estimate the cultural variations in uniqueness quantified by the popularity scheme. Figure 5C shows the quality of the model fit, estimated for $n = 5$ apps, with the model achieving a high $R^2 = 0.7$ explaining a majority of the variance. Figure 5D shows the coefficient estimates with 95% confidence intervals. Remarkably, the median number of apps, which is a proxy for the size of people’s app-fingerprints, is the only informative variable and it is positively associated with country uniqueness values. As the popularity scheme samples apps according to the inverse popularity the larger peoples’ app-fingerprints are the more likely it becomes to pick unpopular apps. This suggests that, on a global scale, there are few options to reduce re-identifiability risks, as regulating the size of app-fingerprints is unfeasible.

**Figure 6:**
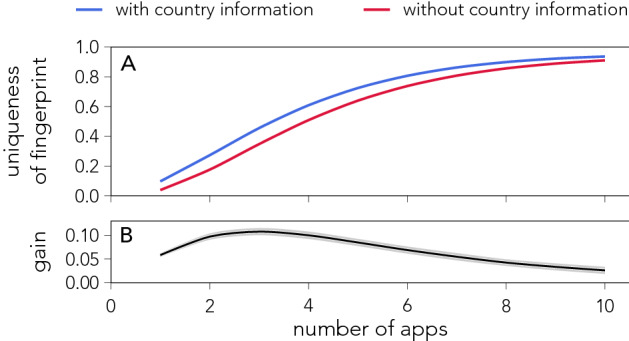
Increased re-identifiability from adding demographic information. (**A**) The average fraction of unique individuals, estimated using the random attack scheme, as a function of the number of apps *n* sampled from the fingerprint, when information about individuals’ country is included (blue line) or not (red line). To exclude effects due to how users are distributed across countries in our dataset, we create a synthetic dataset that includes a random sample of 20,000 individuals from each of the countries under study (see Fig. 4). (**B**) Gain in rate of re-identification when the country information is included. The gain peaks at $n=3$.

Cultural differences, further, open up a new venue of information for de-anonymizing datasets. Adding country of residence as an extra feature in the app-fingerprint makes it easier to re-identify individuals (see Fig. 6A), however, the increase in re-identifiability is not dramatic ($+8.4 \pm 0.5$ percent points for $n=5$) (see Fig. 6B). As such, when limited information is available about app-behaviour, knowledge about an individuals country of residence poses a serious risk to privacy. However, if an individual’s full app-fingerprint is known the country of residence has little significance.

## Discussion

Smartphone behaviour is different from credit card traces and mobility data due to the ease and scale with which behavioural data can be collected, as any app can request permission to access your app history. The economic imperative and the ease of collecting and trading this data at global scale without the users’ knowledge raises serious concerns, especially since this practice has been demonstrated to be in violation of users expectations and knowledge^39,40^. The EU General Data Protection Regulation (GDPR) may be a first step towards addressing these concerns through regulation, since it does mention unicity^41^ and applies globally to data about any EU citizen. In this light, even coarse-grained and binary-level (app/no app) application usage data should be considered personally identifiable information. However, as our study shows, re-identification rates vary significantly depending on the season, highlighting the importance of studying uniqueness across time. As such, privacy policies based on analyses of a single snapshot of user behavior might not provide the adequate protection throughout a year—temporal variability of behavior needs to be considered.

Our study also demonstrates the presence of cultural differences, showing significant variations in uniqueness, even within countries in the European Union. These results opens up for a larger discussion around the impact and portability of policies, as a policy intended to protect users within one country might not provide the same protection for individuals living in a different country. For instance, porting a policy from the US to Europe, or vice versa, might not have the intended effect for the new context. Based on the results from our model, we expect knowledge about the properties of a country app ecosystems, especially the distributions of popularity, to lead to better informed policies for protecting user privacy. We hope our study can bring more nuance to the field of privacy.

This study was performed using app usage data collected from Android phones from a single vendor only. As phone vendor specific apps were disregarded in the analysis, we expect the results to generalize across all Android devices. Further, we have no reason to believe that app usage behaviour and uniqueness are fundamentally different for individuals using iOS devices compared to Android users. iOS has measures in place to protect users from apps that collect app usage for fingerprinting purposes, but these have been shown to be inadequate^42^.

## Methods

### The dataset

We use a dataset that spans 12 months, from Feb. 1st 2016 to Feb. 1st 2017, and contains monthly app-fingerprints for 3,556,083 individuals with pseudonymized app and user identifiers. Each fingerprint is a binary vector composed of the apps a person has used during a month. We do not consider apps that are installed but not used. We further disregard phone vendor specific apps such as: alarm clock, phone dialer, settings etc. and only focus on apps that are downloadable from Google Play. This removes vendor bias, and makes re-identification harder. The users are selected from major markets in Europe, the Americas, and Asia. In total, the number of unique apps in the dataset is 1,129,110, and each individual in the dataset uses at least 3 apps per month. App usage data was collected on Sony Mobile devices as part of the general usage statistics for optimization in order to improve user experience and was processed internally at Sony Mobile. Data collection was approved by the Sony Mobile Logging Board and written consent in electronic form has been obtained for all study participants according to the Sony Mobile Application Terms of Service and the Sony Mobile Privacy Policy. Only countries for which at least 20,000 users accepted the terms of service of the study are included. Due to privacy concerns raw data cannot be shared publicly, however, a data sample of app-fingerprints from 20,000 randomly selected individual, including the hashed id of the apps used in a given month can be found at 10.11583/DTU.13650797.v1. Additionally, we offer the possibility to reproduce our results by spending a research visit at Sony Mobile Communications.

### Estimating uniqueness

To estimate the uniqueness of app-fingerprints, we apply the unicity framework^11^ on *s* samples of 10,000 randomly selected individuals. For each individual we select *n* apps (without replacement) from their app-fingerprint. With the popularity based attack, apps with low user base are selected to increase the uniqueness of the app usage pattern. The person is then said to be unique if they are the only individual in the dataset whose app-fingerprint contains those apps. In cases where *n* is larger the the total length of a person’s app-fingerprint we instead select $\min (n,|\text {fingerprint}|)$ number of apps. Uniqueness for a sample $s_i$ is then estimated as the fraction of the users that have unique traces. Overall uniqueness is the average of the *s* samples, and error-bars are given by the standard deviation. We use $s=20$. To study cultural differences, we apply the unicity framework above on $s=60$ samples of 20,000 randomly selected individuals from the same country (see Supplementary Information for other sample sizes).

### Model of cultural differences

We model dependencies between country unicity and app-ecosystem dependent and control variables as a linear model. To explore monotonic dependencies which not necessary are linear, we rank transform all variables, such that rank 1 is the highest possible value of each variable. The model is defined as $U = \beta _0 + \beta X + \epsilon$, where X is a matrix of the rank transformed variables, $\beta _0$ is the intercept, and $\epsilon$ denotes the residuals (see SI section 3.1 for details). To understand the uncertainty of our analysis, we bootstrap over 10,000 samples to get 95% confidence intervals and report the average $R^2$ value over the samples. We build separate models for the random and popularity attack schemes using $n = 5$ apps.

## Supplementary Information

Supplementary Information.
