# Supplementary material for: Temporal and cultural limits of privacy in smartphone app usage
Source: Sci Rep. 2021 Feb 16;11:3861. doi: 10.1038/s41598-021-82294-1 (PMC7887199; doi:10.1038/s41598-021-82294-1)
Supplement: Supplementary file 1 — Supplementary Information. [file 41598_2021_82294_MOESM1_ESM.pdf]

Temporal and Cultural Limits of Privacy in Smartphone App Usage *Supplementary Information*

Vedran Sekara, Laura Alessandretti, Enys Mones & Håkan Jonsson

S1 The dataset

We use a dataset that spans 12 months, from Feb. 1st 2016 to Feb. 1st 2017, and contains monthly app-fingerprints for 3,556,083 individuals. Each fingerprint is a binary vector composed of the apps a person has used during a month. We do not consider apps that are installed but unused.

We further disregard phone vendor specific apps such as: alarm clock, phone dialer, settings etc. and only focus on apps that are downloadable from Google Play. This removes vendor bias, and makes re-identification harder. The users are selected from major markets in the Americas, Europe and Asia. Thus, the impact of regional variations on uniqueness due to local applications is smaller than if we had sampled users from anywhere in the world.

In total, the number of unique apps in the dataset is 1,129,110, and each individual in the dataset uses at least 3 apps per month.

The data was collected using a pre-loaded recommender app on Xperia phones. Data collection is approved by the Sony Mobile Logging Board and written consent in electronic form has been obtained for all study participants according to the Sony Mobile Application Terms of Service and the Sony Mobile Privacy Policy.

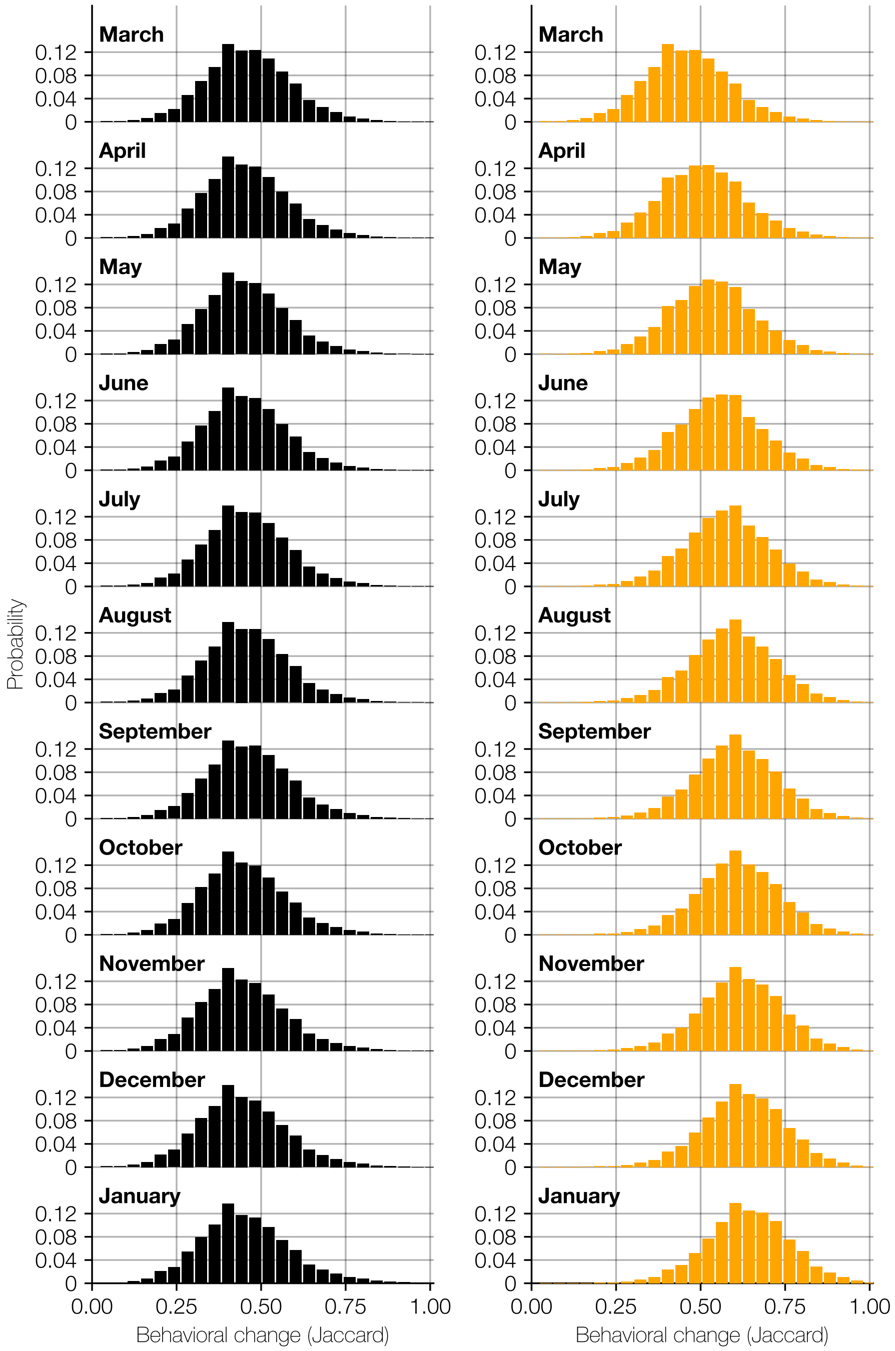

Figure S1: *Distributions show the change in app fingerprint over time. The change is measured as Jaccard distance between a users fingerprint in one month and the next. Left, change between consecutive months, e.g. February and March (denoted March), March and April (denoted April), etc. Right, Difference between fingerprint in February 2016 compared to other months, indicating a drift over time.*

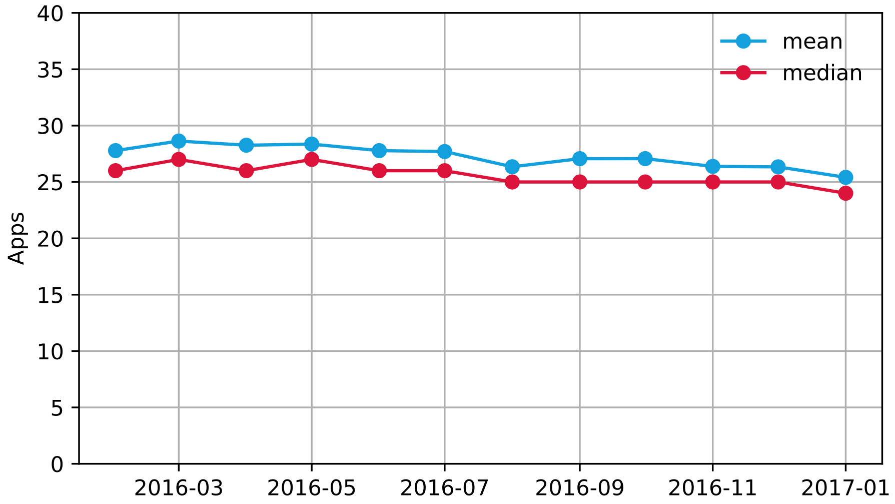

Figure S2: *Average number of apps per user per month. The median is also plotted for comparison.*

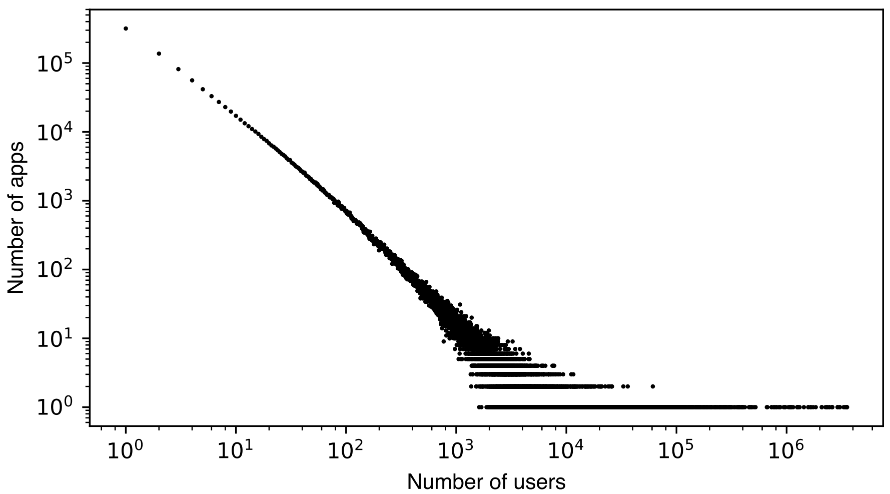

Figure S3: *Distribution of popularity of apps, i.e. the number of individuals using an app. Estimated across the entire dataset. Distribution clearly displays a long-tail.*

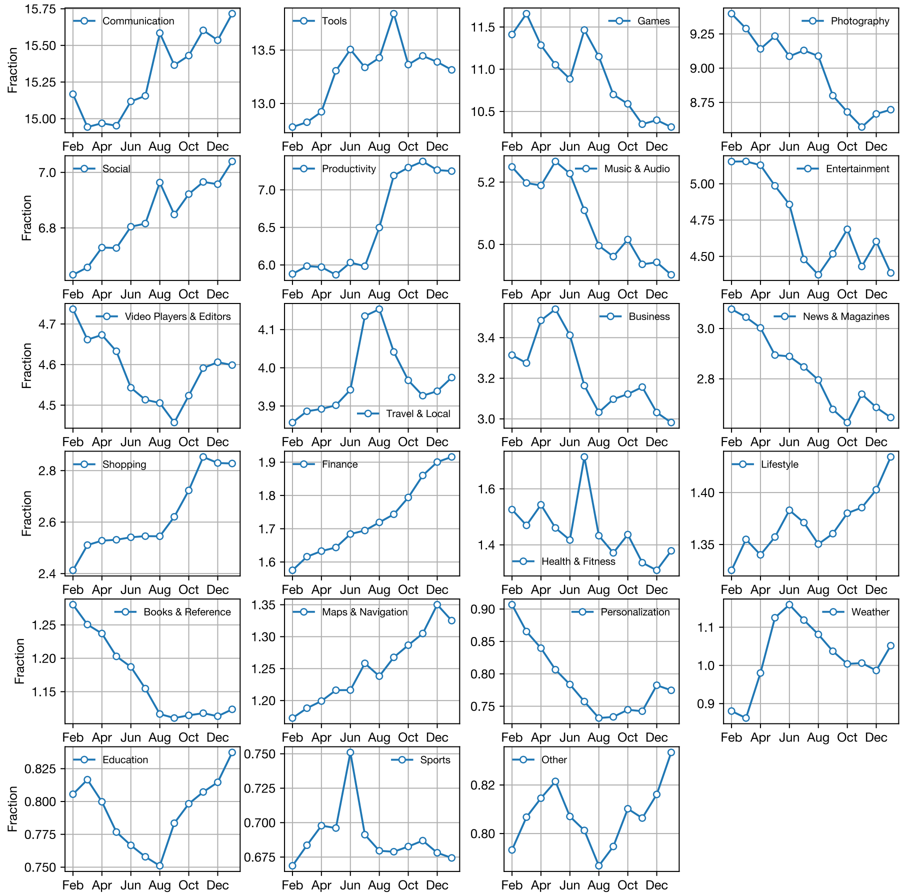

Figure S4: *Fraction of apps per category. Apps are divided into popular Google play categories and figure shows the fraction of app that belong to each category over time.*

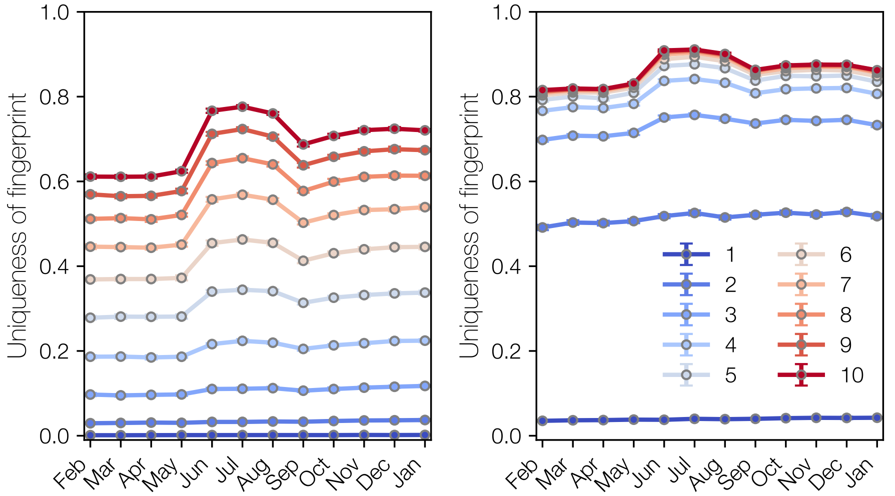

Figure S5: *Seasonable variations of uniqueness over time for the random scheme (left) and the popularity heuristic (right). Because the number of apps people have installed changes over time the curves in Figure 3 (main text) are rescaled. For the random attack scheme we rescale according to* $\widetilde{u}(t) = \frac{u(t)}{|A|_t/|A|_{t=0}}$*, where* $u(t)$ *is the uniqueness at month* t*, and* $|A|_t$ *is the number of apps at time* t*. With* $t = 0$ *denoting the first month of the dataset, February 2016. For the popularity scheme, the curves in the main figure are rescaled according to the probability of picking an app with 100 users or less (*P_t*),* $\widetilde{u}(t) = \frac{u(t)}{P_t/P_{t=0}}$.

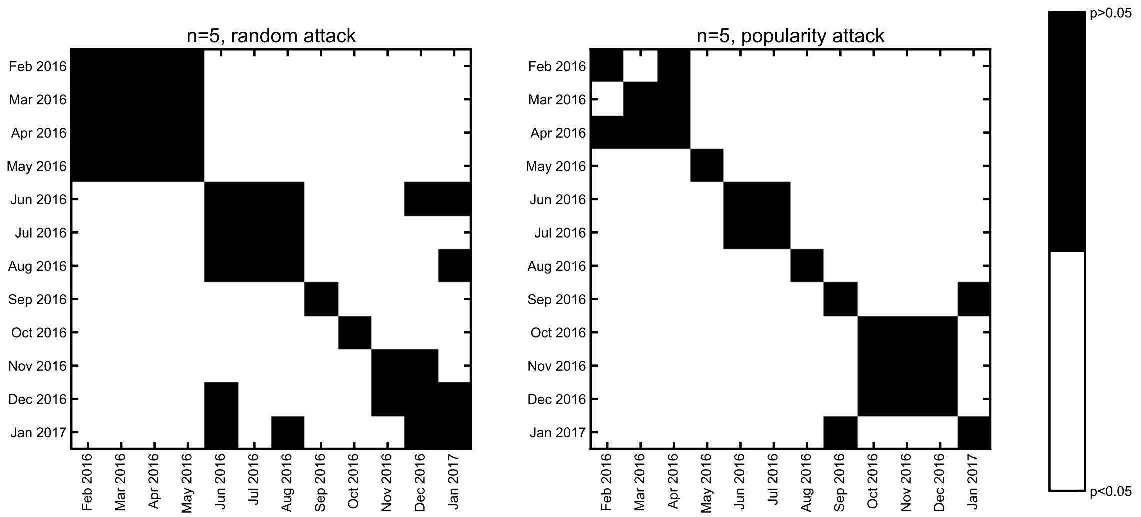

Figure S6: *Differences between months are statistically significant. We compared the distribution of uniqueness between any pair of months across 20 bootstrapping samples using a Welch's t-test with Bonferroni correction for multiple comparisons. The hypothesis that the value of uniqueness is the same for two different months is rejected, with* $p < 0.05$*, for 78% of the months pairs under the random attack scheme and for 89% of the cases under the popularity attack scheme. Results are shown for n=5 apps, for the random (left) and popularity (right) attack schemes.*

S2 Extrapolation to larger population

Subsampling the dataset

To quantify the relation between sample size and uniqueness, we subsample the dataset by selecting a fraction of the original dataset. For each sample s_i we estimate uniqueness using the above methodology. To account for selection bias we estimate uniqueness as the average of multiple realizations of a sample size. We use 20 realizations for sample sizes between 100,000 - 500,000, 10 realizations for samples between 600,000 - 900,000, and 5 realizations for sample sizes above 1,000,000 individuals.

Hiding in the crowd

Our dataset is limited to 3.5 million users, similar in size to a small country, but how will uniqueness change as more users are added (increased sample-size)? Will it become possible to hide in the crowd? More precisely, how does the population size affect the extent to which a specific app-fingerprint remains unique. That is, as more and more users are added to our sample, does the likelihood to observe multiple individuals with identical fingerprints also increase? This corresponds to an inverse k-anonymity problem, where one needs to estimate the number of users that should be added in order to increase the overall anonymity of the dataset. (Bearing in mind that overall anonymity is not a good measure for the sensitivity of individual traces.) To understand the effect of sample-size on unicity, we first slice our dataset into smaller subsamples and use it to estimate the uniqueness for sample sizes ranging from 100,000 to 3.5 million individuals. Figure 4A reveals that sample size has a large effect on the re-identification rate when selecting apps using a random heuristic. Considering $n_{\text{apps}} = 5$, the average re-identification rate decreases from 45.89% for a sample size of 1 million individuals to 37.33% for 2 million individuals and 32.09% for the full sample of 3.5 million people. The attack scheme is considerably less affected (Figure S9). For $n_{\text{apps}} = 5$ we find that the re-identification rates are respectively 96.60%, 94.23% and 92.72% for sample sizes of 1, 2 and 3.5 million individuals. As such, increasing the sample size by 250% (from 1 to 3.5 million individuals) only reduces uniqueness by approximately 4 percent-points.

In order to estimate uniqueness for sample sizes larger than the study population we extrapolate results from Figure S7 for $n_{\text{apps}} = 5$. We express uniqueness of fingerprints using multiple functional forms including: power-laws ($\sim x^{\gamma}$), exponentials ($\sim \exp(\gamma x)$), stretched exponentials ($\sim \exp(x^{\gamma})$), and linear functions ($\sim x$), where x denotes the sample size and γ is a scaling factor. The stretched exponential and power-law show the highest agreement with the data (Figure S9), and roughly suggest that 5 apps are enough to re-identify 75%–80% of individuals for 10 times larger samples (35 million individuals). Although the applied analysis displays high uncertainty with regards to extrapolations, it illustrates the observation that increasing the population size does not help us in hiding in the crowd (that is, uniqueness is not a characteristic of small sample sizes).

Our data sample is not necessarily a representative of the general population, we know that not everybody owns a smartphone and that not everybody downloads custom apps which collect and sell people's data to third parties. More research is needed on this topic,

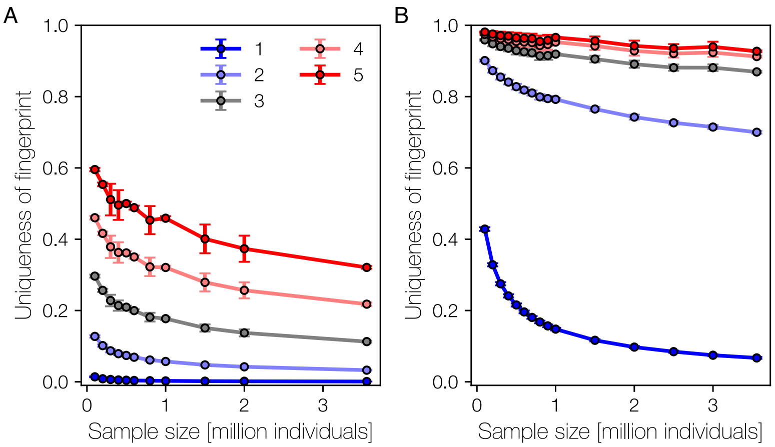

Figure S7: *Identifying fingerprints across data-samples with varying population sizes. Fingerprints are constructed from 12 months of data. The uniqueness of individual fingerprints is reduced (lower re-identification rates) as we increase the sample-size independently of whether apps are selected: (A) randomly or (B) according to the attack heuristic. The magnitude of the change, however, varies greatly different between the two heuristics. Results show in both panels are calculated from multiple realizations of the data (see Materials and Methods section).*

however, our results show that up to 93% (an upper bound) of people from a 30M population might be re-identifiable from 5 apps.

Function	Pseudo R^2	a	b	γ
$ax^\gamma + b$	0.939	-0.031	0.989	0.504
$a\exp(x^\gamma) + b$	0.940	-0.022	1.017	0.261
$a\exp(\gamma x) + b$	0.931	0.066	0.914	-0.388
$ax + b$	0.908	-0.014	0.975	-

Table S1: *Regression values.*

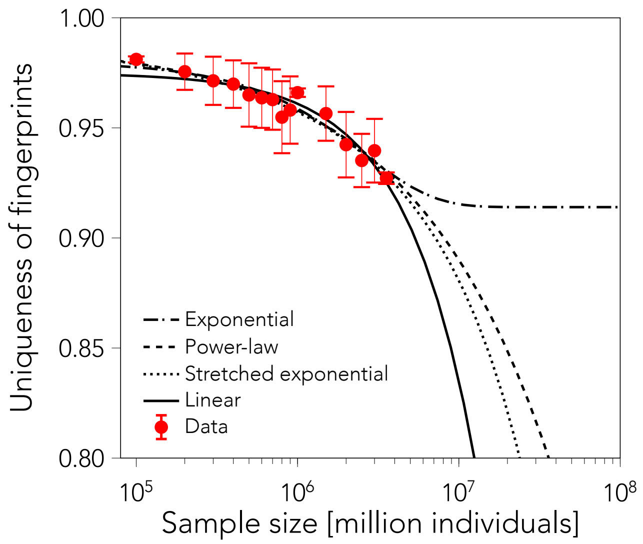

Figure S8: *Extrapolated uniqueness. Fit of different functional forms (see Table S1) to the uniqueness curve for $n_{apps} = 5$ when selecting apps using the popularity heuristic. Closest agreement with data is achieved by the stretched exponential and power law functional forms.*

S3 Country differences

The results presented in the main text on differences between countries are robust with respect to the month of the year, and the sample size chosen. We find that the number of unique individuals, based on a fingerprint of $n = 5$ random apps is substantially stable across 2016 (see Fig. S9). The fit coefficient β characterizing the power-law distribution of app-popularity $P(p)\ p^{-\beta}$ is also stable over time (see Fig. S14). We find that the differences between countries are substantially unchanged for sample sizes of $10,000$ (see Fig. S10), $50,000$ (see Fig. S10) and $20,000$ (see main text Fig 4) individuals.

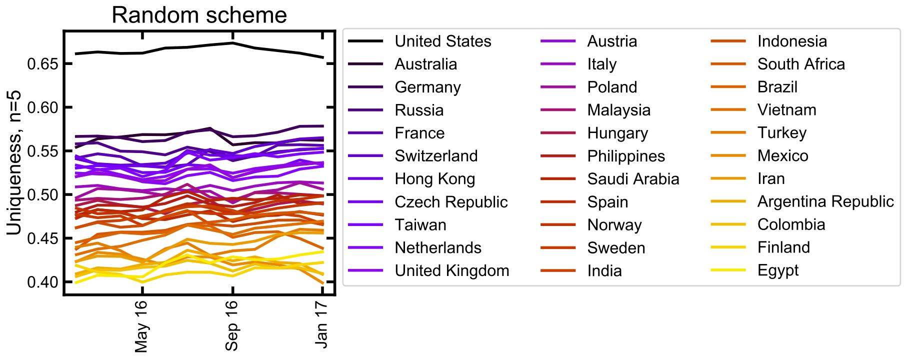

Figure S9: *Country differences are stable in time. The number of unique individuals, based on a fingerprint of $n = 5$ random apps, for different months across 2016. Results are computed for samples of size $20,000$ individuals from the same country.*

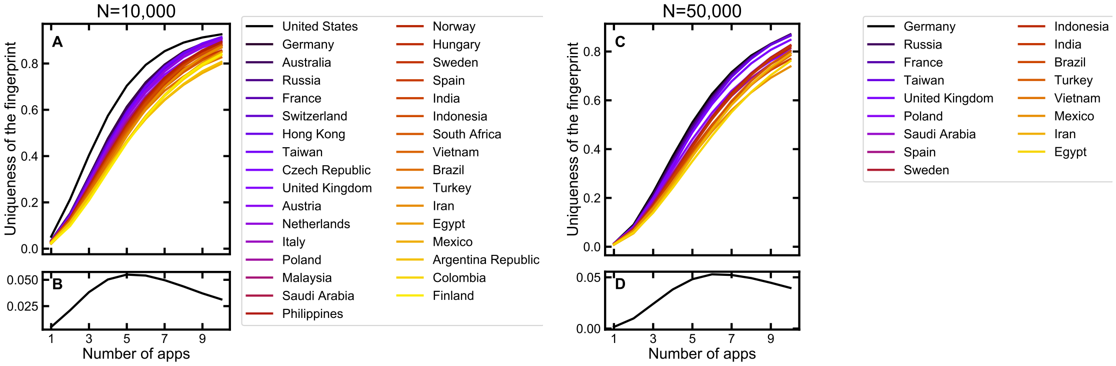

Figure S10: *Differences between countries are robust. (A, C) The average fraction of unique individuals within samples of* $10,000$ *(A) and* $50,000$ *(B) individuals from the same country, as a function of the number of apps n included in the fingerprint. Note that only in a limited number of countries we have more than* $50,000$ *individuals. Results are averaged across* 240 *samples,* 20 *for each of the months considered. Each line correspond to a different country. Countries in the legend are sorted based on the identification rate obtained for* $n = 5$*, from highest to lowest. (B, D) The standard deviation* σ *for the number of unique individuals across countries, as a function of the number of apps included in the fingerprint.*

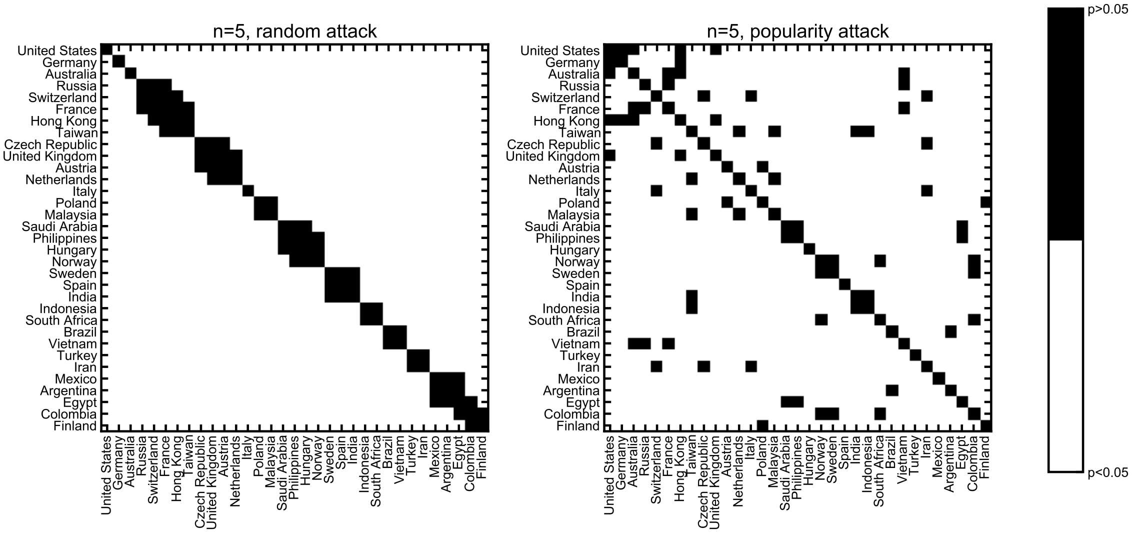

Figure S11: *Differences between countries are statistically significant. We compared the distribution of uniqueness between any pair of countries across 60 bootstrapping samples of* $20,000$ *users using a Welch's t-test with Bonferroni correction for multiple comparisons. The hypothesis that the two countries have the same uniqueness is rejected, with* $p < 0.05$*, for 94% of the pairs under the random attack scheme and for* 92% *of the cases under the popularity attack scheme. Results are shown for the random attack scheme (left, n=5 apps) and the popularity attack scheme (right, n=2 apps).*

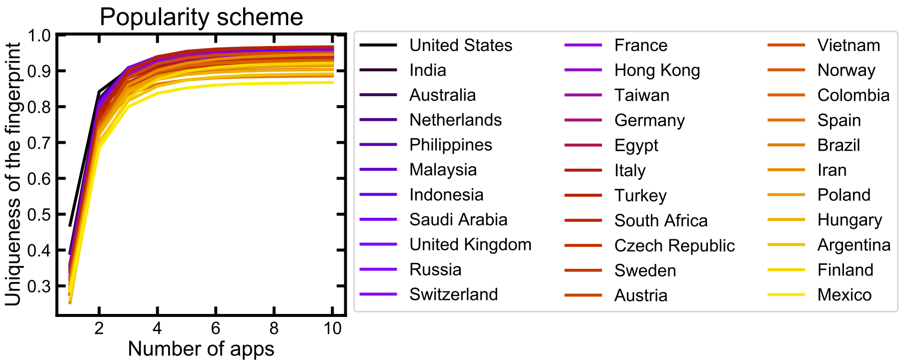

Figure S12: *Country differences when sampling apps using the popularity strategy. Countries in the legend are sorted based on the unicity computed for* $n = 1$.

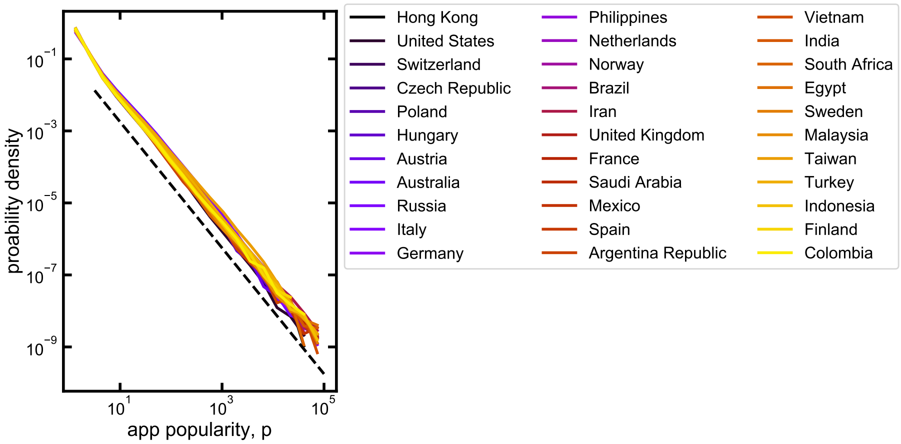

Figure S13: *Different distributions of app popularity across countries. (A) The distribution of app popularity* p*, defined as the fraction of users (in a given country) that have adopted a given app are well described by power laws. Each filled line corresponds to a different country, and the dashed line is a power law* $P(p) \sim p^{-\beta}$*, with* $\beta = 1.75$ *as a guide for the eye. In the legend, countries are sorted based on the corresponding power-law fit coefficient, from highest to smallest. (B) The distribution of app popularity for China (yellow line) and the United States (black line) only.*

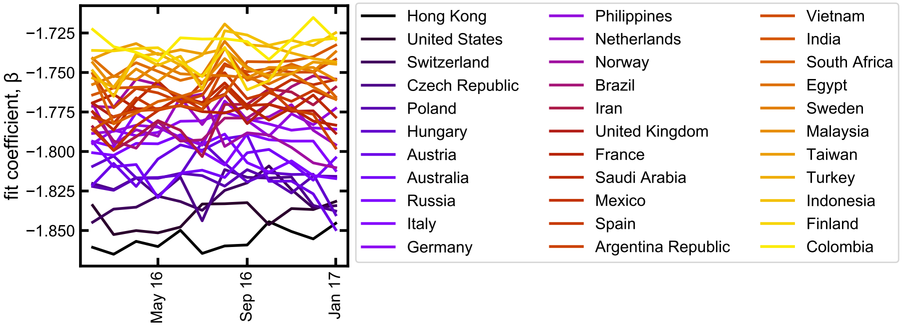

Figure S14: *Fit coefficients are stable in time. The value of the fit coefficient β characterizing the power-law distribution of app-popularity $P(p)$ $p^{-\beta}$ for each month during 2016. Each line is a different country.*

S3.1 Model of cultural differences

To model dependencies between country unicity and app-ecosystem dependent and control variables we use a linear model. To explore monotonic dependencies which not necessary are linear we rank transform all variables, such that rank 1 is the highest possible value of each variable. Initially we considered a larger set of variables relating to the app ecosystem such as number of apps, number of users, ration of apps to users, median number of apps, slope of the app popularity distribution, and fraction of users in population. For the control variables we included population size, GDP per capita (PPP), internet penetration, and the Gini Index of the wealth distribution. However, due to high collinearity between variables we reduced the variable set using a variance inflation factor (VIF) analysis. Fig. S15 shows the correlation plot for the variables in the final model, and variance inflation factors are all below 10.

The model is defined as $U = \beta_0 + \beta X + \epsilon$, where X is a matrix of the rank transformed variables, β_0 is the intercept, and ϵ denotes the residual. We build one model per attack strategy. Fig. S16 shows the normal Q-Q plot and the histogram of the residuals estimated from a for the random strategy, and Table S2 shows the coefficient estimates. For the popularity scheme model Fig. S17 and Table S3, show the residuals and the coefficient estimates. Fig. S18 shows model fits for other values of n apps.

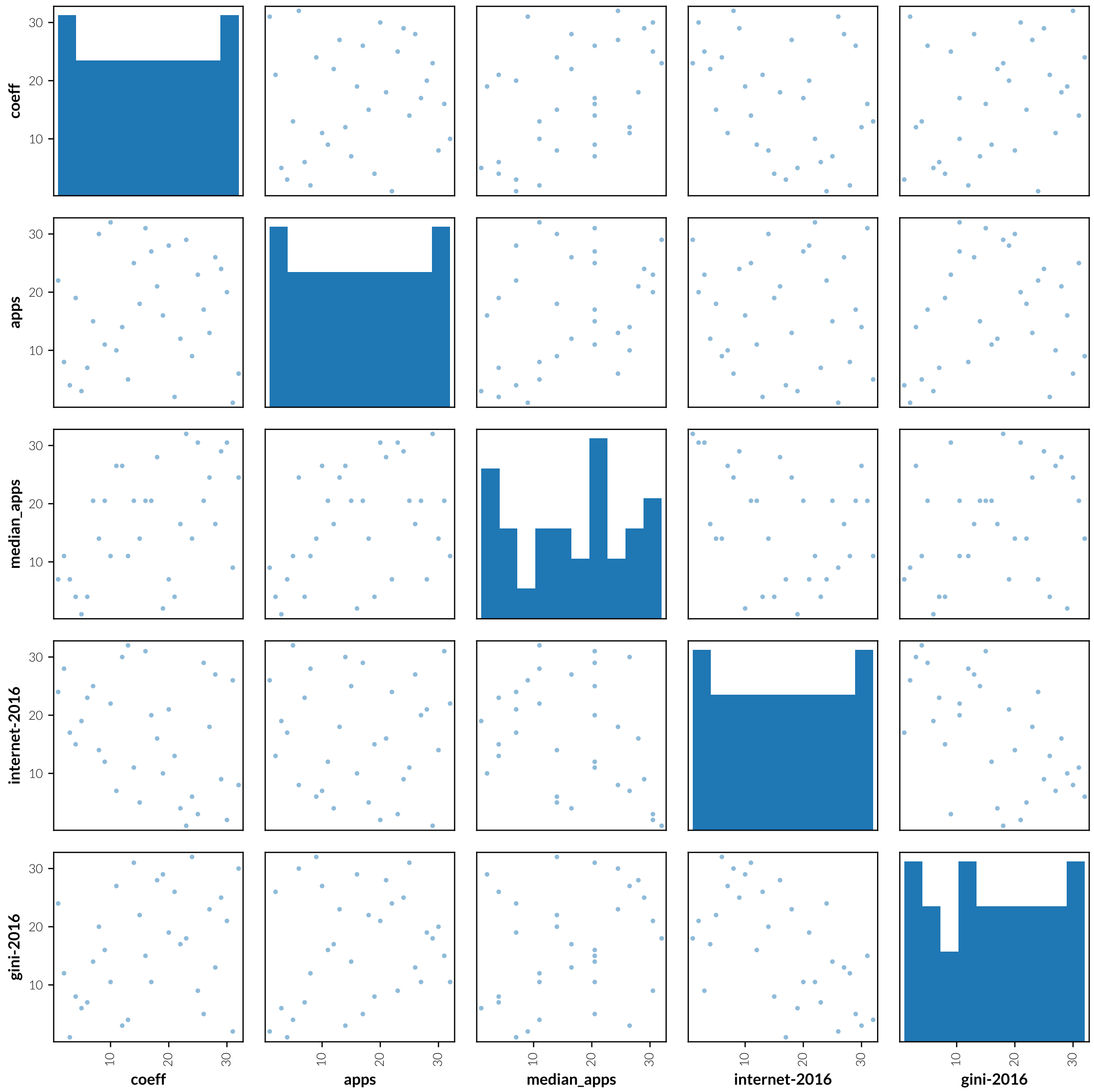

Figure S15: *Correlation plot of all selected variables, illustrating that collinearity is not an issue. Each dot is a country and all variables are rank transformed.*

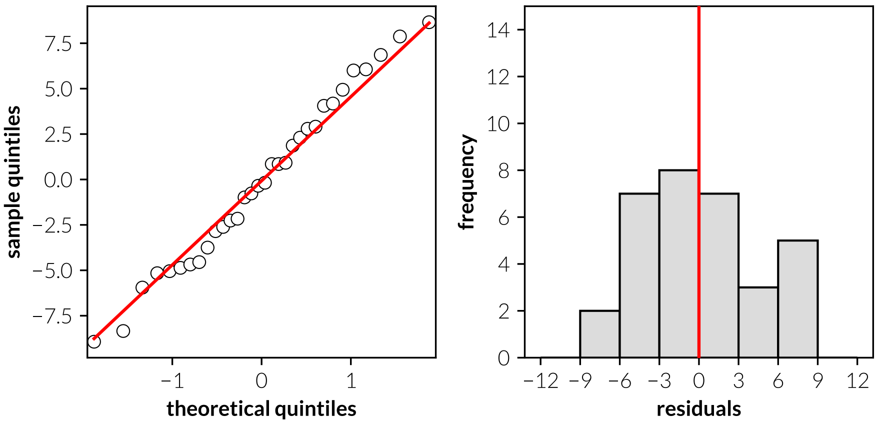

Figure S16: *Residuals for the random scheme model. Left: Q-Q plot of residuals in the model. Right: Histogram of residuals.*

Variable	Mean estimate	95% confidence interval
Intercept	4.26	[-6.11, 13.46]
**Slope of app distribution**	**-0.61**	**[-0.82, -0.37]**
**Number of apps**	**0.29**	**[0.07, 0.50]**
Median apps per person	0.19	[-0.06, 0.41]
**Internet adoption**	**0.46**	**[0.17, 0.74]**
Gini index	-0.03	[-0.33,0.23]

Table S2: *Regression results of the linear model for the random attack scheme with $n = 5$ apps. Confidence intervals are estimated over 10,000 bootstrapped samples.*

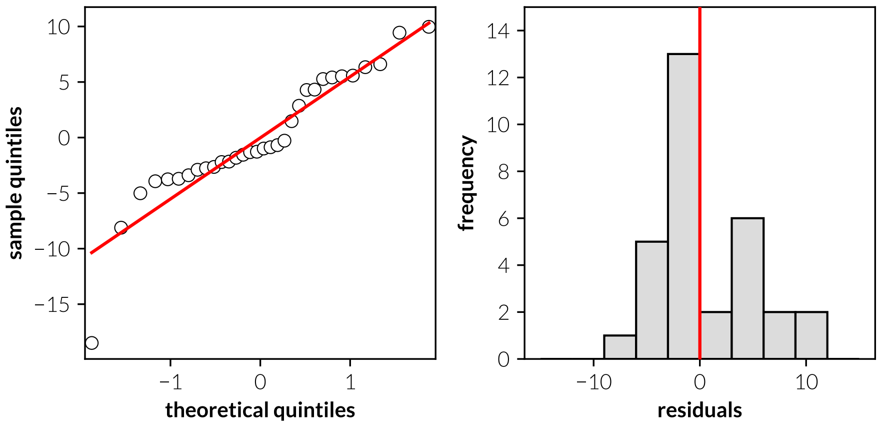

Figure S17: *Residuals for the popularity scheme model. Left: Q-Q plot of residuals in the model. Right: Histogram of residuals.*

Variable	Mean estimate	95% confidence interval
Intercept	4.20	[-5.82, 13.55]
Slope of app distribution	-0.12	[-0.40, 0.22]
Number of apps	0.15	[-0.9, 0.35]
**Median apps per person**	**0.80**	**[0.55, 1.00]**
Internet adoption	-0.02	[-0.30, 0.25]
Gini index	-0.06	[-0.46, 0.27]

Table S3: *Regression results of the linear model for the popularity attack scheme with $n = 5$ apps. Confidence intervals are estimated over 10,000 bootstrapped samples.*

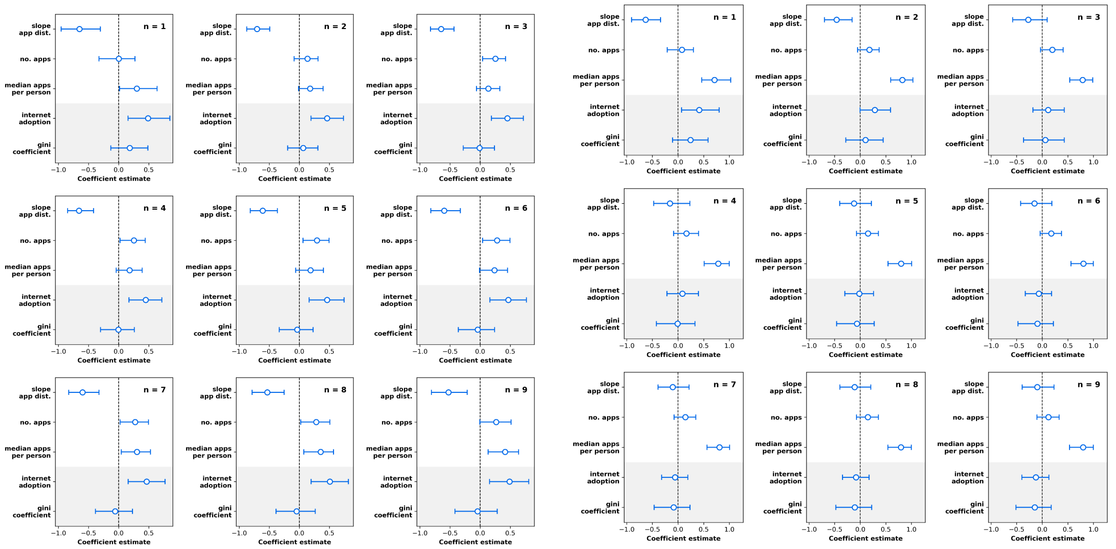

Figure S18: *Coefficient estimates for model fits with different n-values. Left: Estimates for the random attack scheme. Right: Estimates for the popularity scheme. Error-bars denote 95% confidence intervals.*

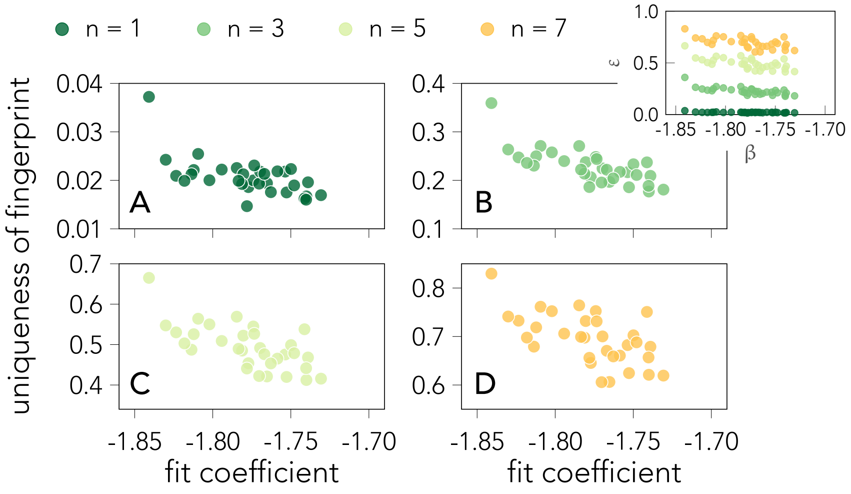

Figure S19: *Negative correlation between the fit coefficient and uniqueness. The number of unique individuals, considering a fingerprint of $n = 1$ (A), $n = 3$ (B), $n = 5$ (C), and $n = 7$ (D) apps versus the fit coefficient β characterizing the power law distribution $P(p) \sim p^{-\beta}$ of app popularity, p. Each dot is a different country.*
